# Mobile Delivery of the Diabetes Prevention Program in People With Prediabetes: Randomized Controlled Trial

**DOI:** 10.2196/17842

**Published:** 2020-07-08

**Authors:** Tatiana Toro-Ramos, Andreas Michaelides, Maria Anton, Zulekha Karim, Leah Kang-Oh, Charalambos Argyrou, Elisavet Loukaidou, Marina M Charitou, Wilson Sze, Joshua D Miller

**Affiliations:** 1 Noom, Inc New York, NY United States; 2 Department of Medicine, Division of Endocrinology & Metabolism Renaissance School of Medicine Stony Brook University Stony Brook, NY United States

**Keywords:** prediabetes, body weight, mHealth, mobile app, mobile phone, randomized controlled trial

## Abstract

**Background:**

The Centers for Disease Control and Prevention (CDC) diabetes prevention program (DPP) has formed the foundation for Type 2 Diabetes Mellitus (T2DM) prevention efforts and lifestyle change modifications in multiple care settings. To our knowledge, no randomized controlled trial has verified the efficacy of a fully mobile version of CDC’s diabetes prevention program (DPP).

**Objective:**

This study aimed to investigate the long-term weight loss and glycemic efficacy of a mobile-delivered DPP compared with a control group receiving usual medical care.

**Methods:**

Adults with prediabetes (N=202) were recruited from a clinic and randomized to either a mobile-delivered, coach-guided DPP (Noom) or a control group that received regular medical care including a paper-based DPP curriculum and no formal intervention. The intervention group learned how to use the Noom program, how to interact with their coach, and the importance of maintaining motivation. They had access to an interactive coach-to-participant interface and group messaging, daily challenges for behavior change, DPP-based education articles, food logging, and automated feedback. Primary outcomes included changes in weight and hemoglobin A_1c_ (HbA_1c_) levels at 6 and 12 months, respectively. Exploratory secondary outcomes included program engagement as a predictor of changes in weight and HbA_1c_ levels.

**Results:**

A total of 202 participants were recruited and randomized into the intervention (n=101) or control group (n=99). In the intention-to-treat (ITT) analyses, changes in the participants’ weight and BMI were significantly different at 6 months between the intervention and control groups, but there was no difference in HbA_1c_ levels (mean difference 0.004%, SE 0.05; *P*=.94). Weight and BMI were lower in the intervention group by −2.64 kg (SE 0.71; *P*<.001) and −0.99 kg/m2 (SE 0.29; *P*=.001), respectively. These differences persisted at 12 months. However, in the analyses that did not involve ITT, program completers achieved a significant weight loss of 5.6% (SE 0.81; *P*<.001) at 6 months, maintaining 4.7% (SE 0.88; *P*<.001) of their weight loss at 12 months. The control group lost −0.15% at 6 months (SE 0.64; *P*=.85) and gained 0.33% (SE 0.70; *P*=.63) at 12 months. Those randomized to the intervention group who did not start the program had no meaningful weight or HbA_1c_ level change, similar to the control group. At 1 year, the intervention group showed a 0.23% reduction in HbA_1c_ levels; those who completed the intervention showed a 0.28% reduction. Those assigned to the control group had a 0.16% reduction in HbA_1c_ levels.

**Conclusions:**

This novel mobile-delivered DPP achieved significant weight loss reductions for up to 1 year compared with usual care. This type of intervention reduces the risk of overt diabetes without the added barriers of in-person interventions.

**Trial Registration:**

ClinicalTrials.gov NCT03865342; https://clinicaltrials.gov/ct2/show/NCT03865342

## Introduction

### Background

In the United States, 84.1 million people are living with prediabetes, and 2 million people are diagnosed with diabetes annually [1]. According to the World Health Organization, an estimated 422 million people worldwide had diabetes in 2014, and the prevalence continues to rise [2]. By 2050, the Centers for Disease Control and Prevention (CDC) estimates that 1 in every 3 people globally will have diabetes [3]. It remains a leading cause of death and disability, accounting for over US $327 billion annually in health care costs [4]. Patients with poor glycemic control develop microvascular complications such as blindness and end-stage renal disease as well as macrovascular complications such as heart attack and stroke [5]. The best way to combat type 2 diabetes mellitus (T2DM) is through prevention.

Restoration of normal glucose regulation in persons with prediabetes decreases the risk of developing T2DM and cardiovascular disease [6]. The most effective intervention to date is the CDC’s diabetes prevention program (DPP) [7]. Studies have demonstrated a modest amount of weight loss through lifestyle modification, with participants significantly reducing their chances of developing T2DM [7-9]. DPP-based educational curricula have been well studied and validated in diverse patient populations, including in-patient settings [10-13]. As a result, findings from the DPP have formed the foundation for T2DM prevention efforts and lifestyle change modifications in multiple care settings.

Prediabetes is often discovered during routine medical visits by way of hemoglobin A_1c_ (HbA_1c_) testing in at-risk individuals (based on the American Diabetes Association [ADA] screening criteria) [14]. Face-to-face time with clinicians is often limited, so adequate delivery of DPP-based initiatives is a challenge. Although clinicians recognize diabetes prevention as an urgent public health need that can dramatically affect the well-being of their patients, a lack of funding, collaboration, and other staff support have been reported to be key obstacles for DPP implementation in clinical practice. In addition, patients have expressed low urgency in seeking further health care after a prediabetes diagnosis [15,16]. Furthermore, highly effective, in-person DPPs can have low participation and adherence [17,18]. Mobile interventions require, or are perceived to require, less commitment, thereby overcoming a barrier in treating those at risk where in-person interventions fall short [18].

Evidence-based, scalable interventions for preventive treatment are urgently needed [16]. Exploring novel ways to empower patients to pursue lifestyle changes to prevent or delay the onset of diabetes is critical in addressing the growing diabetes epidemic. Approximately 4 out of 5 adults in the United States own a smartphone [19], and health information has never been more accessible. As such, it would be desirable to have lower cost, less resource-intensive, and scalable programs that are as effective or superior to in-person programs, especially for people who decline to take part in time-intensive face-to-face programs.

### Previous Work

Studies using mobile-based platforms have focused largely on weight loss rather than diabetes risk reduction. In searching the literature, one study was found that evaluated the efficacy of a mobile-based platform adapted from the DPP curricula based on weight loss in obese patients. However, the sample size was limited, and the HbA_1c_ level was not an end point [20]. Many virtual DPP programs that utilize the internet and social media currently exist and have shown effectiveness similar to the original in-person DPP. However, fully mobile interventions without in-person components that evaluate long-term results have not been tested by means of a randomized controlled trial (RCT). Therefore, this study offers an opportunity to expand our understanding in this area.

Noom (Noom, Inc) is a mobile-based program that delivers structured curricula and coaches who communicate with users in real time through a web-based dashboard. In an observational study, Noom’s DPP program resulted in 85% engagement at the end of the program, in which program completion was defined as having participated in at least one weekly curriculum activity for 9 weeks, per CDC standards and similar to other studies [21]. Upon completion of the core program, participants lost an average 5.6% of body weight [22]. At 65 weeks, the mean weight loss was 6.2% in starters who read one or more lessons per week for ≥4 core weeks, 7.4% in completers who read ≥9 lessons per week on core weeks, and 9.0% in maintenance completers who did any action in postcore weeks (all *P*<.001) [23].

### Objective

The purpose of this RCT was to investigate the effectiveness of a novel, fully mobile, coach-enhanced, DPP program compared with a standard care control with paper-based CDC DPP content. The hypothesis was that participants in the intervention group would have a greater reduction in body weight and HbA_1c_ levels at the study end point after the successful completion of the virtual DPP curriculum.

## Methods

### Recruitment

This parallel RCT took place at Stony Brook Medicine’s tertiary care ambulatory clinics from October 2016 to June 2018 in Long Island, New York. Subjects were recruited from general internal medicine, family medicine, and endocrinology practices. The inclusion criteria included patients who were English speaking, were >18 years old, were a referral from the patient’s physician, had an HbA_1c_ level of 5.7% to 6.4% within 3 months before study enrollment, and owned a smartphone (Apple or Android). The exclusion criteria included patients who had experienced recent weight loss (by patient report, >5 pounds in the 6 months preceding the enrollment visit), had a previous diagnosis of type 1 diabetes mellitus or overt T2DM, had serious or persistent mental illnesses, had >72 hours of hospitalization in the past 30 days, who were currently enrolled in a structured weight loss program or within the month preceding study enrollment, were pregnant or nursing, who had given birth within the past 3 months, and had been discouraged by a physician to enroll in a DPP program.

Participants were identified through the electronic medical record based on prediabetes diagnostic criteria (as defined by the ADA [24]). Clinicians at each clinic site were provided with information describing the study and the designated contact for study enrollment. Study personnel contacted individuals for participation, either in person or by telephone call if they had expressed interest in participating in the study. Potential participants were given an information sheet describing the study before enrollment. After indicating an interest in study participation, informed consent was obtained by the study personnel in person or by telephone. All procedures in this study were in accordance with the ethical standards of the World Medical Association Declaration of Helsinki, and all study protocols were approved by Stony Brook University’s institutional review board. This trial was retrospectively registered with ClinicalTrials.gov under NCT03865342 on March 9, 2019. The Consolidated Standards of Reporting Trials statement is included in Multimedia Appendix 1.

### Randomization

Participants were enrolled and randomized by study coordinators to either the intervention (Noom Coach) or control study arms utilizing a random number generator with a 1:1 allocation ratio, which automatically concealed the previous allocation. The sequence was generated by an external statistician using SAS software, version 9.4, of the SAS system for Windows (SAS Institute Inc). Coordinators set up intervention group participants with the mobile program free of cost and provided control group participants with a printed version of the DPP curriculum [25]. Study coordinators provided Noom with only the first names and email addresses of the randomized participants. No protected health information was provided to Noom by the investigators. Coordinators contacted each participant for the 6- and 12-month visits. Clinicians who gathered follow-up data were unaware of the random allocation, and masking was not broken for the duration of the study.

### Procedures

Subjects were weighed at the offices of their physicians at the time of study enrollment, between 5 and 7 months (6-month time point), and between 11 and 13 months (12-month time point) poststudy enrollment. In-clinic weight measurements were recorded in the electronic medical record as part of usual care at the time point of each visit. Patients who attend these clinics are normally scheduled to visit their physician every 6 months; study participants were not asked by study personnel to see their physician any more frequently than as part of usual care. HbA_1c_ testing was completed at baseline, 6, and 12 months. If at any measurement time, the HbA_1c_ level increased above ADA criteria for overt T2DM, participants were counseled and referred to their primary care provider. The baseline HbA_1c_ level was any HbA_1c_ level available in the electronic medical record for the participant in the 3 months before study enrollment. Primary care and endocrinology clinics used the DCA Vantage (Siemens) point-of-care (POC) HbA_1c_ machine (shown to have high levels of accuracy and precision for HbA_1c_ between 5% and 8%) [26,27]. This is a clinical laboratory improvement amendment–waived capillary fingerstick test to allow clinicians to check patients’ HbA_1c_ levels in the clinic. POC testing is offered to all patients in these practice sites and was offered to study participants at each of their respective HbA_1c_ testing time points. The HbA_1c_ level from commercial laboratory visits within the required period was used in lieu of POC testing when available. Of all HbA_1c_ measurements for the participants in this study, 99% at baseline, 98% at 6 months, and 95% at 12 months originated from commercial laboratory visits. At baseline and 6 months, the POC values were from the intervention group, and at 12 months, they were equally distributed.

As an incentive for participating, subjects were offered US $10.00 gift cards to Starbucks at the 6- and 12-month follow-up visits at the time of weight and HbA_1c_ measurements.

**Figure 1:**
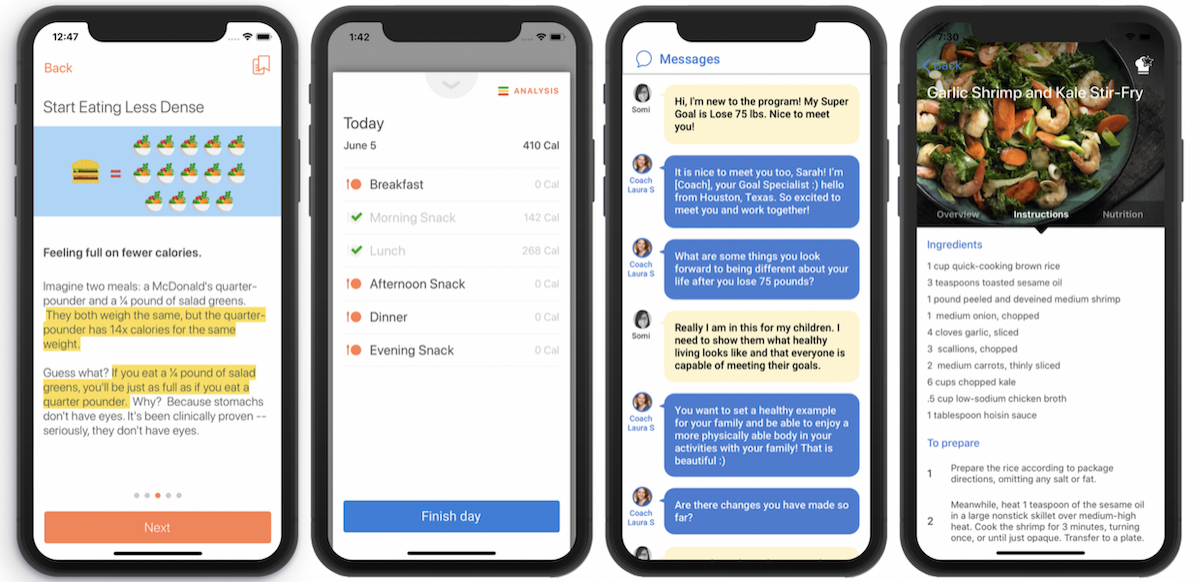
Selection of screen pages for mobile health intervention.

For the intervention group, within the program, an assigned Noom Coach digitally communicated with participants individually and as a group [23]. During the first week of the study, participants randomized to the intervention learned how to use the Noom program, how to interact with their coach, and the importance of maintaining motivation throughout the program. Participants had mobile access to coach-participant messaging, group messaging, daily challenges for behavior change, the DPP education articles (weekly bite-sized content over 20 weeks for the core portion and up to 52 weeks for the maintenance phase), food logging with color coding, steps and exercise logging, and automated feedback based on food choices (Figure 1). They were asked to log their weight by self-report, meals, and physical activity within the program on a weekly basis. National Diabetes Prevention Program–certified coaches securely monitored participant progress through a web-based dashboard. Participants could communicate as needed to support their individual journeys and could expect to hear from their coach every day.

Coaches working with the participants in the Noom program were trained to meet National Diabetes Prevention Program standards and trained in motivational interviewing techniques. Motivational interviewing is a client-centered therapeutic modality that utilizes positive regard, reflections, and the illumination of client strengths as a route to behavior change [28]. Coaches assisted users in setting specific, measurable, attainable, realistic, and time-based goals on a weekly basis. Coaching functions as a productive addition to a weight loss intervention through the use of accountability, feedback (food logs and choices), problem-solving, and positive reinforcement for the desirable behavior [29]. Motivational interviewing has previously shown promising results in weight loss in women with T2DM [30]. This increase in weight loss for those receiving motivational interviewing was proposed to function through treatment adherence.

### Outcomes

Primary outcome measures included a change in weight and HbA_1c_ levels at 6 and 12 months from the start of the program. The CDC’s National Diabetes Prevention Program benchmark for weight loss is ≥5% at 6 and 12 months [1]. Secondary outcomes were exploratory and included in-program actions (indicators of program engagement) as predictors of the change in weight and HbA_1c_ levels. Any known occurrence of serious adverse events in study participants, defined as death, serious violent incidents, and formal complaints about the intervention were recorded.

The engagement categories were measured by weekly numbers of logged meals, logged weigh-ins, logged steps, articles read, posts in the group, and messages to the coach. Previous research has suggested that focusing on specific areas of behavioral regulation of food intake [31], physical activity [31], education [32,33], coaching [29], and self-monitoring of weight through regular weigh-ins [34] may promote behavior and lifestyle changes and further weight loss success.

### Statistical Analysis

The sample size was determined using the estimated SDs of the change in HbA_1c_ levels from an intervention study of patients with prediabetes (N=129) [35]. Using an SD of 0.36 and α of .05 resulted in a final sample of 224 study participants (112 per group) at 80% power to identify a minimum detectable difference of 0.5% in change in HbA_1c_ levels.

Descriptive statistics were calculated for baseline characteristics, including mean, SD, and 95% CI, to summarize differences from baseline to 6 months and to study the conclusion at 12 months between program intervention and control groups. Linear mixed models tested the null hypothesis that the mean weight and HbA_1c_ levels of the intervention and control groups were equal over time, after adjusting for other covariates. Hypothesis tests are two-sided at the .05 significance level. Multiple linear regression examined in-app actions and engagement variables as predictors of weight loss in the intervention group. Changes in weight and HbA_1c_ levels in the intervention group were analyzed first regardless of program completion. As program engagement is a key factor in attaining clinically meaningful outcomes, we further conducted prespecified analyses based on participants who completed the program as per CDC standards [36]. Maximum likelihood estimates with estimation maximization algorithms were used for missing data. All statistical analyses were performed using SPSS Statistics for Windows (version 21.0; IBM Corp), Minitab (version 17.0; Minitab, LLC), and Mplus (version 8.1; Muthén and Muthén).

## Results

### Participant and Study Characteristics

**Figure 2:**
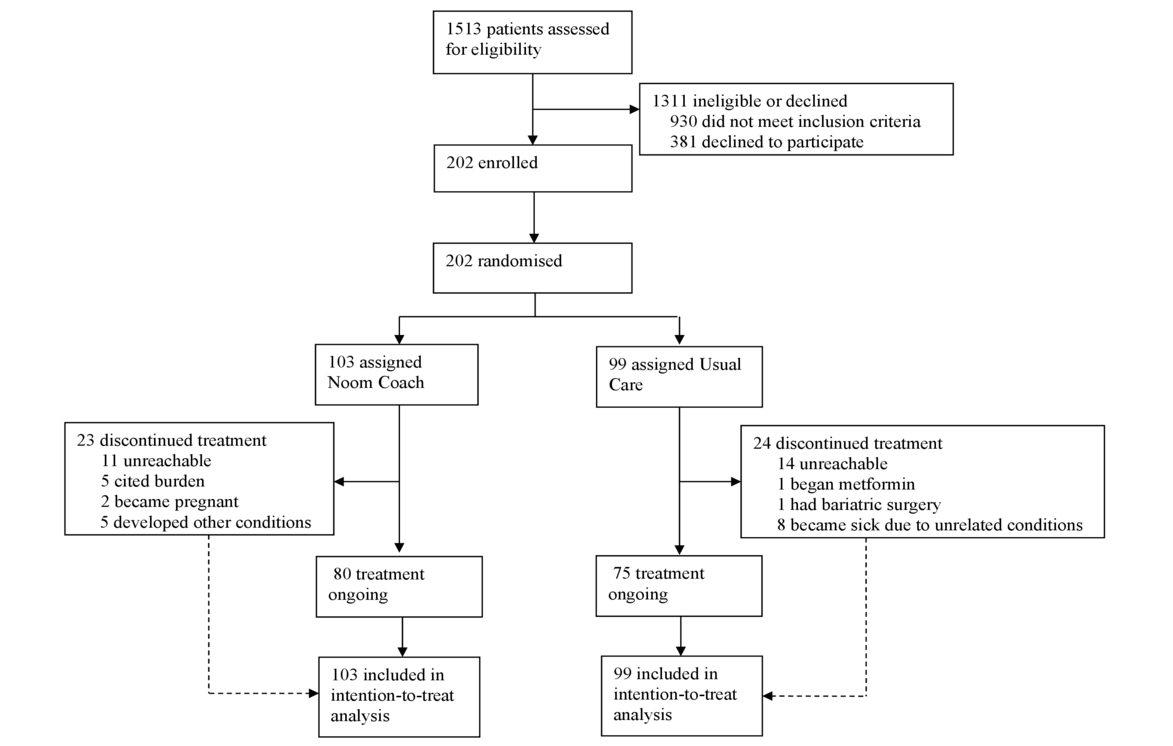
Trial profile of recruitment and completion.

Between October 2016 and June 2017, 1513 potential participants were assessed for eligibility, of whom 930 did not meet the inclusion criteria and 381 declined to participate. From this, 202 participants who met the study criteria were recruited and randomized (Figure 2). Participation and retention were high, with 82.2% (166/202) of participants completing all study follow-up visits. At baseline, no differences were observed between groups in weight, BMI, HbA_1c_ level, or demographic characteristics (Table 1). Among the participants, 73.8% (76/103) of the intervention and 69% (68/99) of the control group were female, with a BMI of 31.3 kg/m^2^ and 30.9 kg/m^2^, respectively. The mean age was 55.7 years for the intervention group and 57.5 years for the control group. No study-related serious adverse events were reported.

**Table 1:** Baseline characteristics of the study groups.

Demographic characteristics	Intervention group (n=103)	Control group (n=99)
Women, n (%)	76 (73.8)	68 (69)
Weight (kg), mean (SD)	85.71 (21.47)	85.93 (22.02)
Hemoglobin A_1c_ (%), mean (SD)	5.94 (0.18)	5.93 (0.19)
Height (m), mean (SD)	1.66 (0.08)	1.66 (0.09)
BMI (kg/m^2^), mean (SD)	31.25 (6.43)	30.94 (7.23)
Age (years), mean (SD)	55.69 (13.63)	57.54 (12.45)

Among 80 intervention participants who downloaded the program, 27 did not engage meaningfully (completed fewer than 4 in-app actions, for example, read less than one article per week over 4 weeks), 53 started (logged an action and read articles for at least four weeks), and 45 completed the program (logged an action and read articles for at least nine weeks). At 6 months, among those who had weight data, 31% (28/91) of the intervention group (if they started the program or not) and 38% (17/45) of completers lost >5% body weight compared with 14% (11/77) in the control group. At 12 months, among those who had weight data, 27% (25/91) in the intervention group and 38% (17/45) of completers maintained >5% body weight loss compared with 14% (10/72) in the control group.

Similar to the control group, no significant changes in weight or HbA_1c_ levels were seen in those who did not engage meaningfully or start the program. Weight and HbA_1c_ levels by intention-to-treat (ITT) and by completion at 6 and 12 months are shown in Multimedia Appendix 2.

### Weight and Hemoglobin A_1c_ Changes at 6 Months

In the ITT analyses, accounting for missing data, changes in weight and BMI were significantly different at 6 months between the intervention and control groups. Weight and BMI were lower in the intervention group at 6 months by −2.64 kg (SE 0.71; *P*<.001) and −0.99 kg/m^2^ (SE 0.29; *P<*.001), respectively. No difference was seen in HbA_1c_ levels between groups at 6 months (mean difference 0.004%, SE 0.05; *P*=.94).

**Figure 3:**
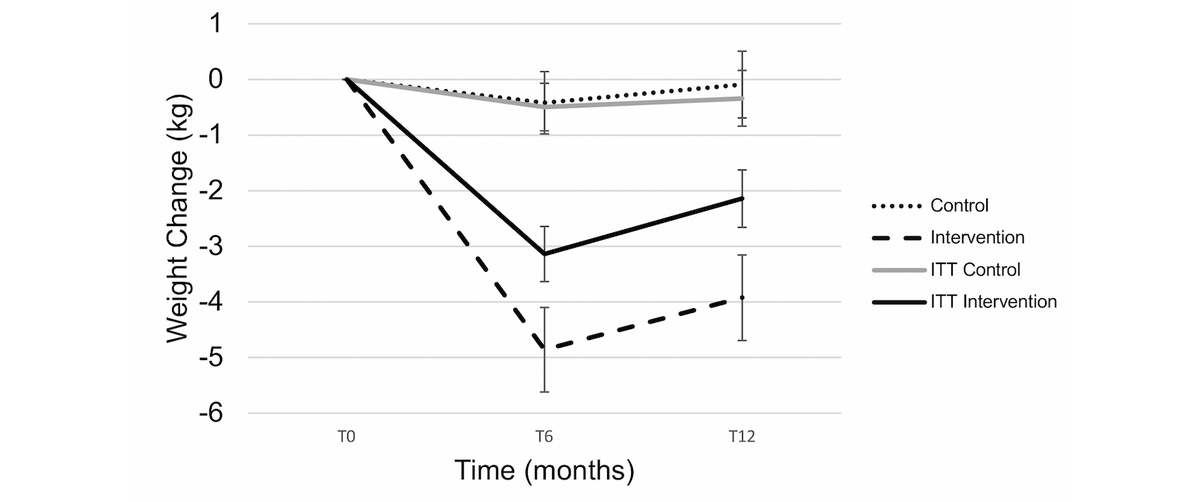
Weight change across time points. ITT: intention-to-treat; T0: baseline; T6: 6 months; T12: 12 months. ITT: intention-to-treat; T0: baseline; T6: 6 months; T12: 12 months.

**Figure 4:**
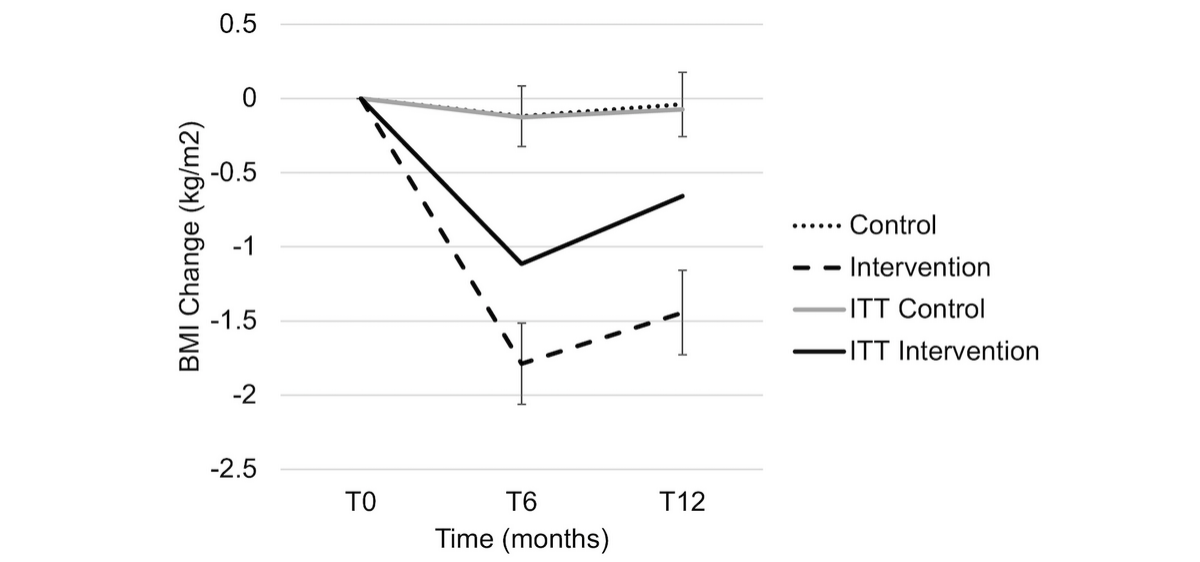
BMI change across time points. ITT: intention-to-treat; T0: baseline; T6: 6 months; T12: 12 months.

**Figure 5:**
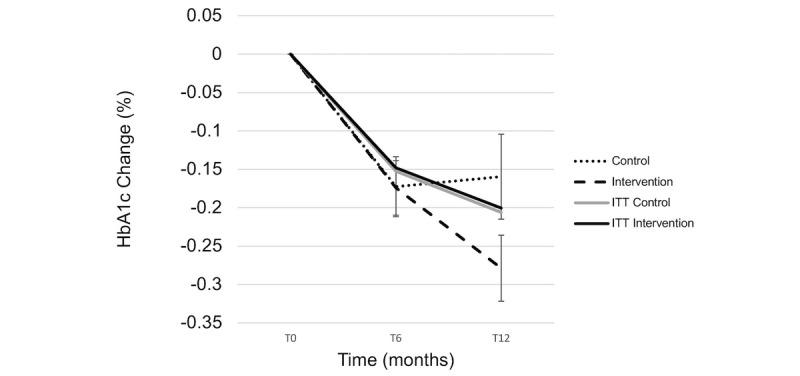
Hemoglobin A_1c_ change across time points. ITT: intention-to-treat; T0: baseline; T6: 6 months; T12: 12 months.

In the analysis that did not involve ITT, weight loss was significant over time in the intervention group but not in the control group, and there was a significant interaction of group by time (*P*=.04), indicating that weight loss was dependent on being in the intervention group. Those who completed the program achieved a clinically and statistically significant weight loss of 5.6% at 6 months based on paired *t* tests (Table 2; Figure 3). BMI significantly decreased over time in the intervention completers group versus the control group (*P*<.001; Figure 4). The HbA_1c_ levels significantly decreased over time in both groups at 6 months, but there was no significant interaction of group by time (*P*=.35; Figure 5). In the intervention group, those who completed the program did not report a HbA_1c_ level above 6.4% at any time point. In the control group, 2 patients reached HbA_1c_ levels at or above 6.4% at 6 months.

**Table 2:** Change in body weight and hemoglobin A_1c_ at 6 and 12 months by participant group (intervention, intervention completers, and control group).

Measured values	Intervention group^a^						Intervention completers						Control group^a^			
	6 months, mean (95% CI)	Effect size^b^	*P* value^c^	12 months, mean (95% CI)	Effect size^b^	*P* value^c^	6 months, mean (95% CI)	Effect size^b^	*P* value^c^	12 months, mean (95% CI)	Effect size^b^	*P* value^c^	6 months, mean (95% CI)	*P* value^c^	12 months, mean (95% CI)	*P* value^c^
Weight (kg)	−3.31 (−4.43 to −2.19)	−0.12	<.001	−2.22 (−3.31 to −1.13)	0.01	.02	−4.86 (−6.39 to −3.33)	−0.22	<.001	−3.92 (−5.48 to −2.37)	−0.14	<.001	−0.42 (−1.53 to 0.69)	.45	−0.09 (−1.30 to 1.11)	.88
Weight (%)	−3.69 (−4.89 to −2.48)	−0.63	<.001	−2.54 (−3.74 to −1.33)	−0.40	<.001	−5.59 (−7.22 to −3.95)	−0.99	<.001	−4.66 (−6.42 to −2.90)	−0.72	<.001	−0.15 (−1.42 to 1.11)	.81	0.33 (−1.06 to 1.72)	.63
BMI (kg/m^2^)	−1.35 (−1.79 to −0.92)	−0.13	<.001	−0.88 (−1.31 to 0.44)	−0.00	<.001	−1.79 (−2.34 to −1.24)	−0.15	<.001	−1.44 (−2.02 to −0.87)	−0.09	<.001	−0.12 (−0.53 to 0.29)	.56	−0.04 (−0.47 to 0.39)	.86
HbA_1c_^d^ (%)	−0.15 (−0.22 to −0.08)	0.08	<.001	−0.23 (−0.32 to −0.14)	−0.13	<.001	−0.17 (−0.25 to −0.10)	−0.06	<.001	−0.28 (−0.37 to −0.19)	−0.38	<.001	−0.17 (−0.25 to −0.09)	<.001	−0.16 (−0.27 to −0.05)	.01

^a^At 6 months, the intervention group lost 2.64 kg more and had a BMI difference of 0.99 kg/m^2^ compared with the control group (both *P*<.001) in the intention-to-treat (ITT) maximum likelihood estimates missing data analyses. At 12 months, in the ITT analyses, the intervention group lost 1.8 kg more than the control group (*P*=.01) and had a BMI difference of 0.58 kg/m^2^ (*P*=.01). In the ITT analyses, HbA_1c_ was not different between groups at 6 or 12 months, with a difference of 0.004% (*P*=.94) and 0.006% (*P*=.93), respectively.

^b^≤0.2=small effect; >0.2 to <0.8=medium effect; ≥0.8=large effect.

^c^*P* values were obtained from *t* tests using a .05 significance level.

^d^HbA_1c_: hemoglobin A_1c_.

### Weight and Hemoglobin A_1c_ Changes at 12 Months

In the ITT analyses, changes in weight and BMI were significantly lower in the intervention group at 12 months by −1.80 kg (SE 0.81; *P*=.01) and −0.58 kg/m^2^ (SE 0.24; *P*=.01), respectively. HbA_1c_ levels showed no difference between the groups at 12 months (0.006%; SE 0.07; *P*=.93).

In the analysis that did not involve ITT, an interaction between group and time was found (*P*<.001), indicating that weight loss was dependent on being in the intervention group. Those who completed the program achieved a clinically and statistically significant weight loss of 4.7% at 12 months (Table 2; Figure 3). BMI significantly decreased over time in the intervention completers group versus the control group at 12 months (*P*<.001; Figure 4). At 12 months, the HbA_1c_ levels continued to significantly decrease in the intervention group, which was below the prediabetic values (Figure 5). In the intervention group, those who completed the program did not report HbA_1c_ levels above 6.4%. In the control group, 4 patients reached HbA_1c_ levels at or above 6.4% at 6 months.

### Engagement Variables

Completers actively participated in the program (Table 3). Multiple linear regressions controlling for age, sex, baseline BMI (and baseline HbA_1c_ levels for weight change models) observed which program engagement behaviors, including the mean number of weekly weigh-ins, articles read, meals logged, steps, group posts, and messages to coach predicted changes in weight and HbA_1c_ levels at 6 and 12 months. Weight change at 6 and 12 months was also evaluated as a predictor in the models for HbA_1c_ levels change at 6 and 12 months, respectively.

Having a higher baseline BMI and HbA_1c_ status predicted increased weight loss at 6 months (Table 4). Important program engagement predictors of weight loss at 12 months included frequency of weighing-in (β=−0.30; *P*=.01) and logging more steps (β=−0.21; *P*=.08). Weight change at 12 months was predicted by higher meal logging frequency (β=−0.41; *P*=.001). In-app actions did not predict a change in HbA_1c_ levels at 6 and 12 months.

**Table 3:** Engagement of intervention participants (in-app actions per week as).

In-app activities	Intervention, mean (SD)		Completers, mean (SD)	
	6 months	12 months	6 months	12 months
Logged meals^a^	7.40 (8.03)	5.01 (6.43)	12.58 (6.73)	8.53 (6.39)
Logged weigh-ins^b^	0.42 (0.83)	0.29 (0.72)	0.70 (1.0)	0.48 (0.89)
Logged steps	12,132 (14,131)	9487 (12,494)	19,110 (14,479)	15,152 (13,511)
Articles read	3.92 (5.90)	2.28 (3.80)	6.62 (6.48)	3.86 (4.33)
Group comments^c^	0.14 (0.32)	0.07 (0.17)	0.23 (0.40)	0.11 (0.20)
Messages to coach	1.44 (1.91)	0.94 (1.46)	2.42 (1.98)	1.58 (1.63)

^a^Logged meals refers to the times breakfast, lunch, snack, and dinner were logged per week.

^b^Logged weigh-ins refers to times per week of in-app weight self-reports.

^c^Group comments refers to responses to group posts per week.

**Table 4:** Multiple linear regression models for in-app actions as predictors of body weight change adjusting for age and gender.

Dependent variable and significant predictors		Beta level	*t* value^a^	*P* value	*r* ^2^	Adjusted *r*^2^	*F* value^a^
**Weight change at 6 months**							
	BMI	−0.302	−2.676	.01	0.21	0.24	6.158^b^
	HbA_1c_^c^	−0.305	−2.765	.01	0.21	0.24	6.158
	Steps at 6 months	−0.205	−1.815	.08	0.21	0.24	6.158
	Weigh-ins at 6 months	−0.296	−2.628	.01	0.21	0.24	6.158
**Weight change at 12 months**							
	Meals at 12 months	−0.405	−3.603	.001	0.16	0.15	12.983^d^

^a^The *df* values for *t* test and *F* test were unavailable.

^b^*P*≤.001.

^c^HbA_1c_: hemoglobin A_1c_.

^d^*P*=.001.

## Discussion

### Principal Findings

This RCT shows that a fully mobile DPP intervention with coaching in adults with prediabetes was effective in significantly reducing weight over 1 year. In the ITT analyses, the intervention group lost 2.64 kg more at 6 months and 1.80 kg more at 12 months compared with the control group, but the HbA_1c_ level was not significantly different between groups over time. Weight loss has been shown to plateau at a 5% to 9% loss 6 months into a program with a slight weight regain, indicating a 4.8% to 8% loss by 12 months with approximately a 3% to 4% loss maintained at 48 months [37]. Our data corroborate these findings in that slight weight regain was seen between 6 and 12 months, with intervention participants not returning to baseline weight.

In the analyses that did not involve ITT, participants in the intervention group who completed the program lost 5.6% and 4.7% body weight at 6 and 12 months, respectively, compared with the control group that had no meaningful weight change. In the analyses that did not involve ITT, HbA_1c_ levels were reduced in both groups at 6 months, but only the intervention group continued to significantly decrease through 12 months. Higher program engagement predicted greater weight loss but not a change in HbA_1c_ levels. To our knowledge, this is the first such RCT demonstrating long-term efficacy for weight loss in a fully mobile DPP intervention.

### Comparison With Previous Work

Diabetes prevention interventions have been delivered through multiple means, including in-person, community, the web and mobile, and mixed interventions [35,38-42], reporting weight loss of 3% to 5% or more. In 2009, a web-based DPP intervention showed that completers lost 4.79 kg at 12 months [42]. To our knowledge, this study is the first to verify the longitudinal efficacy through an RCT of a fully mobile-based DPP encompassing all aspects of a CDC recognized program [43]. It adds to the growing digital DPP intervention literature by demonstrating a clear, ITT reduction in weight at 1 year using a modern state-of-the-art digital mobile health program in a relatively pragmatic setting.

We previously showed significant weight loss in a fully mobile DPP observational study at 24 and 65 weeks [22,23], with program completers achieving higher degrees of weight loss compared with nonstarters and starters. Similar to a web-based DPP intervention with −4.7 kg and −4.0 kg weight loss at 6 and 12 months, respectively, in those who completed the program [44], we found −4.9 kg and −4.0 kg at 6 and 12 months in the intervention completers. Another DPP intervention of older adults found that more participants completed a web-based DPP program compared with an in-person intervention and achieved 5% weight loss over 6 and 12 months [45].

A higher baseline BMI and HbA_1c_ level, logging more steps, and more frequent weigh-ins predicted greater weight loss at 6 months, whereas continuing to log meals at 12 months predicted higher weight loss at 1 year. Previously, weighing-in and logging more meals along with group interaction predicted weight loss at 24 weeks [22], whereas at 65 weeks, meal logging and interacting with a support group continued to be significant weight loss predictors, supporting self-monitoring as a key component of successful weight loss and maintenance [23]. Indeed, social support in the form of in-app group interaction and high self-efficacy evidenced by persistent food logging are crucial factors that predict weight loss success, further supporting the design of personalized intervention strategies through mobile health (mHealth) [46]. Those with higher HbA_1c_ levels had greater reductions in weight, which might imply that borderline HbA_1c_ levels should not necessarily prompt clinicians to prescribe medication before referring them to a DPP, helping reduce the development of overt T2DM without the need for medication. Weight change at 6 or 12 months was not a predictor of HbA_1c_ change, indicating that a larger sample and longer study duration may be needed to establish a stronger association.

This study demonstrates that a control group receiving usual care plus the CDC’s DPP written materials did not lead to meaningful weight loss or reduction in HbA_1c_ levels. Previous mobile-based prediabetes and weight loss interventions have shown efficacy, and here we offer further evidence of the feasibility of a fully mobile-based DPP intervention leading to meaningful sustained weight loss for up to 1 year. The efficacy of this approach is clinically significant because it is likely that a larger number of people at risk can participate in such an intervention as smartphone technology removes the barriers of time and accessibility presented by in-person interventions. Mobile interventions can easily be adapted by medical professionals to facilitate patient participation and engagement in DPP programs to reduce T2DM risk and advance patient care while improving their practice’s efficiency.

### Limitations

Program participation was a limitation of this study. We found that 23 participants declined to download the program or redeem the intervention program’s registration code despite remaining enrolled in the study and completing most visits. Another 53 participants did not meaningfully engage with or complete the program. Designing interventions with protocols to re-engage lapsed study participants may improve participation.

The smartphone version of the DPP allows for intensive education that can be disseminated over time due to the various program features for a variety of forms of communication. Studies have shown that patient engagement and diabetes risk awareness are poor [47,48]. Low self-motivation may impair timely treatment, pointing to the added need for intensive education to create true awareness and understanding of the prediabetes condition and potential benefits of a DPP. These factors seem to be significant gaps in clinical care and reporting suboptimal adoption of behaviors for risk reduction in adults with prediabetes [48]. Such findings suggest the need to improve patient education and awareness in the clinical setting to prevent the onset of T2DM and decrease the risk of future complications.

Despite the ability to measure if participants went through the educational content in the program, there was no way to ensure that they read the content fully or engaged with activities or practices that were proposed to be done off of the app. Another limitation of our study is that individuals with prediabetes with a BMI <25 kg/m^2^ were included in our analyses, reducing the effect size of weight loss and HbA_1c_ levels. Additionally, although 82% of participants completed all study visits, the study remained underpowered for HbA_1c_ outcomes. A larger sample size would have provided a more robust estimate of the effectiveness of HbA_1c_ levels. Future research is needed to establish wider generalizability and true efficacy for HbA_1c_ reduction resulting from the program intervention.

Despite these limitations, this study is the first mHealth DPP RCT from a program fully recognized by the CDC showing significant long-term weight loss greater than 5% and maintenance up to 1 year. Furthermore, completers in the intervention group had a large enough weight loss that drove significant ITT weight loss. The main outcome measures were reliably obtained in a controlled clinical setting, removing potential measurement errors that can arise through self-reporting. Study participants were predominantly female, which might limit generalizability; however, the sample was recruited from tertiary care outpatient medical practices representative of the general population at risk for T2DM on Long Island. Furthermore, the sample included a broad range of ages ≥18 years and individuals with normal BMI, indicating that intervention findings could apply to the general at-risk population.

Individuals who are more aware of a medical condition or risk are more likely to seek additional medical care and significant lifestyle changes. The perception that body weight poses a health risk to the individual significantly contributes to their efforts to lose weight [49]. People who have been told by physicians that they are at a health risk if they do not change their weight could have more active program engagement than others, as this advice has been shown to be strongly related to weight loss efforts [49,50]. Future research will focus on testing brief but intensive prediabetes education sessions after diagnosis and before referral to a DPP to aid in a better understanding of the true risks faced with such a diagnosis. A brief preparatory intervention can result in higher uptake of in-person, mobile, web-based, or combination DPPs and effectively prevent T2DM and improve health outcomes in more persons who may have not sought care.

### Conclusions

As the first long-term randomized intervention of its kind, the results of this study demonstrate that a novel fully mobile-based smartphone-delivered DPP with human coaching is an effective and powerful tool for attaining clinically and statistically significant weight loss up to 1 year, reducing T2DM risk as well as in-person interventions but without the added barriers. Continuous guidance through coaching, seamless self-monitoring tools, and engaging mobile-delivered DPP content are key to achieving sustained changes in weight and preventing T2DM.

## Appendix

Multimedia Appendix 1 Consolidated Standards of Reporting Trials checklist.

Multimedia Appendix 2 Body weight and hemoglobin A1c at 6 and 12 months by participant group.

Multimedia Appendix 3 CONSORT-EHEALTH checklist (V 1.6.1).

## Abbreviations

ADA: American Diabetes Association
CDC: Centers for Disease Control and Prevention
DPP: diabetes prevention program
HbA_1c_: hemoglobin A_1c_
ITT: intention-to-treat
mHealth: mobile health
POC: point-of-care
RCT: randomized controlled trial
T2DM: type 2 diabetes mellitus
