# Supplementary material for: Mobile Delivery of the Diabetes Prevention Program in People With Prediabetes: Randomized Controlled Trial
Source: JMIR Mhealth Uhealth. 2020 Jul 8;8(7):e17842. doi: 10.2196/17842 (PMC7381044; doi:10.2196/17842)
Supplement: Multimedia Appendix 2 [file mhealth_v8i7e17842_app2.docx]

**Table 2**. Body weight and HbA1c at 6 and 12 months by participant group (mean ± SD)

|  | n | ITT^a^ Intervention | n | Intervention | n | Completers | n | Non-Starters | n | ITT Control | n | Control |
| --- | --- | --- | --- | --- | --- | --- | --- | --- | --- | --- | --- | --- |
| Weight (kg) | 103 | 85.86 ± 21.59 | 103 | 85.40 ± 21.59 | 45 | 85.20 ± 17.99 | 58 | 85.57 ± 24.17 | 98 | 85.86 ± 22.02 | 98 | 85.93 ± 22.02 |
| 6-month weight (kg) | 103 | 82.72 ± 20.87 | 88 | 82.23 ± 19.55 | 45 | 80.34 ± 16.71 | 43 | 84.21 ± 22.17 | 98 | 85.36 ± 21.28 | 77 | 84.75 ± 21.69 |
| 12-month weight (kg) | 103 | 83.72 ± 21.66 | 91 | 84.21 ± 22.22 | 45 | 81.27 ± 17.84 | 46 | 87.09 ± 25.69 | 98 | 85.52 ± 21.24 | 72 | 84.30 ± 19.64 |
| HbA_1c_ (%) | 103 | 5.94 ± 0.18 | 102 | 5.94 ± 0.18 | 45 | 5.92 ± 0.17 | 57 | 5.96 ± 0.20 | 98 | 5.94 ± 0.19 | 99 | 5.93 ± 0.19 |
| 6-month HbA_1c_ (%) | 103 | 5.79 ± 0.27 | 62 | 5.82 ± 0.30 | 31 | 5.78 ± 0.20 | 31 | 5.86 ± 0.38 | 98 | 5.78 ± 0.26 | 62 | 5.80 ± 0.31 |
| 12-month HbA_1c_ (%) | 103 | 5.74 ± 0.41 | 74 | 5.73 ± 0.44 | 33 | 5.65 ± 0.22 | 41 | 5.80 ± 0.56 | 98 | 5.73 ± 21.25 | 57 | 5.79 ± 0.42 |

^a^ ITT: Intention to treat
